# Optimum Clearance in the Microblanking of Thin Foil of Austenitic Stainless Steel JIS SUS304 Studied from Shear Cut Surface and Punch Load

**DOI:** 10.3390/ma13030678

**Published:** 2020-02-03

**Authors:** Yohei Suzuki, Ming Yang, Masao Murakawa

**Affiliations:** 1 Komatsuseiki Kosakusho Co., Ltd., 942-2 Shiga, Suwa, Nagano 392-0012, Japan; 2 Tokyo Metropolitan University, 6-6 Asahigaoka, Hino, Tokyo 191-0065, Japan; yang@tmu.ac.jp; 3 Nippon Institute of Technology, 4-1 Gakuenndai, Miyashiro, Minamisaitama, Saitama 345-8501, Japan; masa.murakawa@gmail.com

**Keywords:** punch load, cut surface quality, optimum clearance, fine blanking, blanking experimental, finite element method analysis

## Abstract

An extrusion-type fine blanking with a negative clearance was proposed by the authors instead of standard fine blanking for creating a full-sheared surface in the micro blanking process. In this study, micro blanking experiments and finite element analyses with narrow, zero and negative clearances are carried out for the optimizing the clearance at which a shear cut surface can be finished with a full-sheared surface with the minimized punch load. Fracture criterion, hydrostatic stress and maximum punch stress for the conditions with various clearances are investigated. As a result, it was clarified that the clearance at which the cut surface does not fracture and minimization of the punch load is achieved is gained by the use of clearance −4 μm.

## 1. Introduction

In recent years, attention has been paid to micro-processing technologies suitable for mass production. However, problems, such as the lowering of material forming limits due to thinner materials [1,2,3,4] and the lowering of dimensional accuracy (e.g., the fracture that occurs on the cut surface during shearing) due to the downsizing of product dimensions, have not been solved. Various research studies have been conducted on manufacturing methods for the microfabrication of small parts, such as laser processing, etching processing, and electron beam processing [5,6,7]. However, these microfabrication methods are not necessarily optimal from the viewpoint of productivity and cost. The authors have selected press stamping, which is excellent in terms of both productivity and cost, and examined the feasibility of a micropart manufacturing method. More specifically, an involute tooth profile part with a microsize and a complicated shape was selected as a representative example of a microsize part, and the so-called fine blanking (FB) [8,9] for the effect of hydrostatic pressure on the ductility of the metal [10], was used. In particular, the feasibilities of the finish-type FB using the narrow clearance, and the extrusion-type FB using the negative clearance, were verified, and the relationship between the difference between the narrow and negative clearances and the shear cut surface was investigated. The results revealed that it was possible to process full-sheared surfaces by adopting negative clearance [11]. In addition, the finite element method (FEM) and material crystal analysis by Electron Back Scatter Diffraction (EBSD) have also revealed the difference in the processing mechanism between the two FB methods [12].

However, according to the above-mentioned verification results, it was predicted that the load applied to the punch tip would be increased by adopting negative clearance. This suggests that, in general, punch breakage and wear will also increase [13]. Even in conventional shearing, punch breakage and wear are two of the most important issues, and various studies on punch load in conventional shearing have been conducted so far. For example, Aoki et al. studied the tool cutting edge wear mechanism by shear experiments [13], Maeda et al. verified the progress of the tool cutting edge wear [14], Koga et al. verified the relationship between clearance and tool wear [15], and the punch wear and quality of punched products, in punching thin sheets [16,17,18,19,20].

FE analysis has been used to elucidate various processing mechanisms, and it has been also used for punch load analysis in blanking. For example, Nakashima et al. obtained an equivalent stress on the tool by analyzing the axisymmetric model with the tool set to an elastic body [21], and Hambi reported tool wear results in press stamping using a finite element code wear prediction model [22]. Falconnet et al. reported punch wear in the blanking of a copper thin sheet [23,24]. However, the punch load due to the difference in negative clearance, including zero clearance in the FB proposed in previous reports [11,12], has not been verified or analyzed. In this study, we aim to derive the optimal clearance at which the shear cut surface can be finished with a full-sheared surface and the punch load can be minimized by experimental verification and finite element analysis at the narrow, zero, and negative clearances.

## 2. Experimental Procedure

### 2.1. Blanking Condition

**Figure 1:**
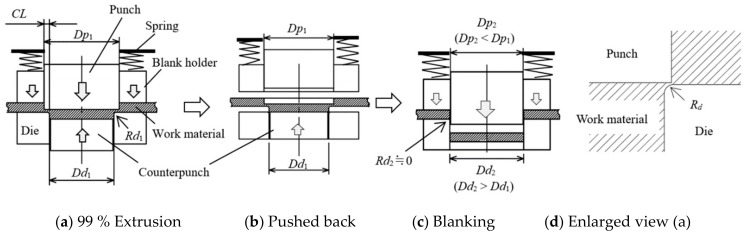
Schematic of extrusion blanking used in the mass-production progressive die system [11].

From the blanking experiment at the narrow clearance, zero clearance, and negative clearance, the conditions under which the cut surface does not fracture were verified. At the same time, the load during blanking at each clearance was measured. The material to be processed was JIS SUS304 made by TOKUSHU KINZOKU EXCEL Co., Ltd. (Tokyo, Japan), with a plate thickness *t* of 0.1 mm and a width of 20 mm. Table 1 shows the mechanical properties of the material. Table 2 shows the material of tools’ (punch and die) composition and mechanical properties, available from Fuji Die Co., Ltd. (Tokyo, Japan) Next, Figure 1 shows a schematic diagram of the blanking process using negative clearance developed for progressive machining in actual production to explicitly explain how negative clearance punching is technically feasible, and Table 3 shows the detailed specifications of the die comprehensive blanking experiment series, particularly from the viewpoint of various tool clearances. To explain from another viewpoint, of the aforementioned comprehensive experiment series, referring first to die-set production, the punch is processed, using a grinding machine for small to micro-precision tools and high-precision production parts, and then the die is roughly processed by a wire cutting process and finally lapped to remove the deformed superficial layer on the tooth surface. In addition, to secure the positional accuracy of the tool (punch/die), JIS-SKD11, which can ensure strength and dimensional stability against heat treatment, can be used for parts such as the stripper plate, punch plate, and die plate. Jig-grinding finish was used for drilling the relevant parts where a relative position relationship is required to align the punch and the die and, additionally, shim tape was used to move the die position in 1 μm unit. For blanking experiment series, a cemented carbide punch with *Dp_1_* = φ1.748 mm was combined with a cemented carbide die with an inner diameter (*Dd_1_*), such that the clearance (*CL)* values between the punch and the die were 2, 0, −2, −4, and −8 μm (2%*t*, 0%*t*, −2%*t*, −4%*t*, −8%*t*) respectively. At a positive clearance *CL*, a punch stroke greater than the plate thickness of the workpiece is technically feasible, as aforementioned with reference to Figure 1, but at zero and negative clearances, if the punch stroke exceeds the plate thickness, the punch and die could come into contact with each other. Strictly speaking, since the die radius corner has actually a radius value of 0.01 mm, the punch and die do not come into contact even if the punch stroke becomes equal to the plate thickness. Therefore, in the actual process, the punch stroke was stopped once it was at 99% of the plate thickness, and the remaining 1% was made into a progressive die structure that could be removed at the next stage of the die set. The die edge was provided with a small radius portion of R and a counter punch to suppress cracking during shearing. The plate presser force was set to a maximum of about 500 N using a coil spring, and the reverse presser force was set to a maximum of about 200 N by the same method. For measuring the punch load, a load cell was provided on the upper surface of the punch, and a load displacement diagram was obtained by measuring the slide displacement of the press plate with a laser displacement meter. The press machine itself was not exclusively designed for FB purposes; a general-purpose screw servo press machine (made by DT-J515 Microfabrication Research Laboratory with pressurization capacity of 50 kN) was used, in order to have the same functions. It should be stressed here that controlling the amount of punch stroke is important to prevent interference between the punch and the die. In this experiment, the slide displacement of the press machine, measured with a laser displacement meter, and a load cell set on the upper surface of the punch, could be controlled in an amount of 1 μm unit.

### 2.2. FEM Simulation Model and Conditions

**Figure 2:**
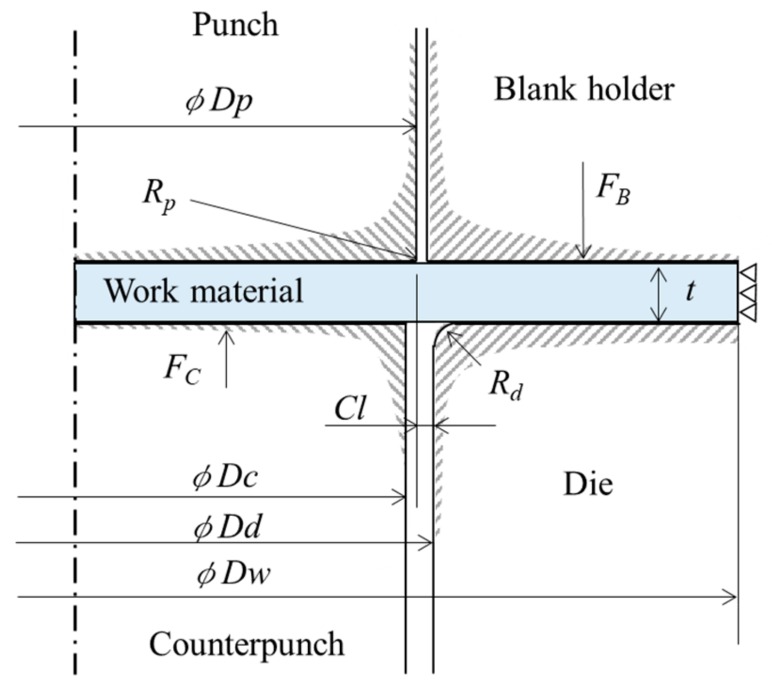
Finite Element Method simulation model (axisymmetric model).

**Figure 3:**
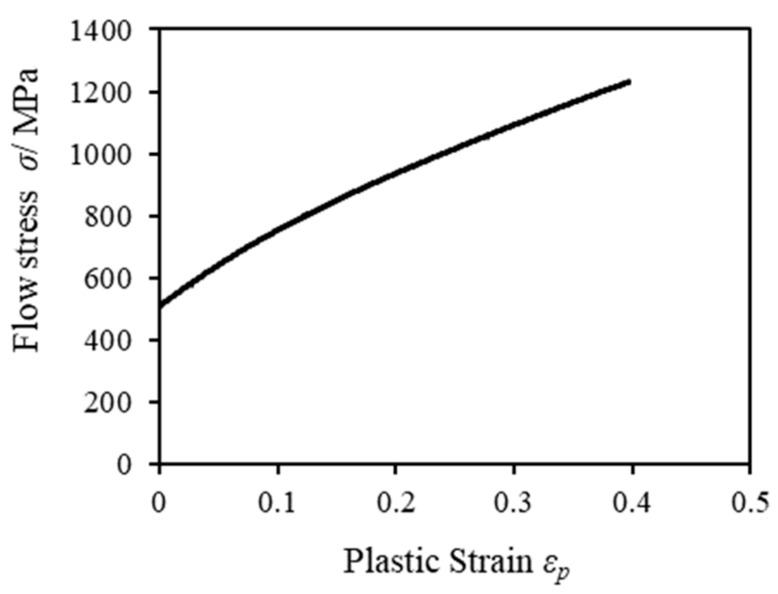
Flow stress–plastic strain curve.

Evaluation of the cut surface and punch load at each clearance, obtained by the aforementioned comprehensive blanking experiments, were considered by the following three FE analysis methods. The first utilized the ductile fracture condition value (hereinafter referred to as damage value *C*) from the maximum tensile/compressive principal stress generated in the shearing region and evaluated and compared the cut surfaces from the blanking experiment result and the FE analysis result. The second evaluation compared hydrostatic stress, which is one of the important parameters of FB. The third evaluation compared the punch stress between samples. Figure 2 shows the employed finite element simulation model, and Table 4 shows the FEM simulation conditions for this model. The analysis model was axisymmetric, with the analysis time taken into consideration, and the commercial code DEFORM2D (Version 11.3) was used. The punch and die are assumed to be elastic bodies, and the number of elements is set to about 10,000. The blank holder/stripper and counter punch are assumed to be rigid bodies. The work material is assumed to be elastoplastic, and the number of elements is set to about 15,000. Four-node rectangular elements were generated on the tools and the work material. As is known by the skilled persons in the shearing industry, in the deformation region around the tool edge, the deformation is concentrated in a very narrow range, and a large distortion occurs in the elements. Therefore, mesh size was determined by referring to the simulation results [25] in the previous FB study, which showed the relationship between the mesh size and the clearance *CL*. Specifically, the mesh size was set to about 1 μm, which is smaller than the clearance. To prevent the interruption of analysis when an element is more deformed than the certain condition during the analysis, a remeshing function for reproducing the element available from the relevant FE code was applied. The remeshing condition was based on the possible depth of interference between the work material element and the tool boundary (specifically, this interference depth was 1 μm). Incidentally, the aforementioned FEM conditions were based on the fact that the results of the blanking experiment and the FEM analysis were in good agreement in the previous report [12]. For the punch and die, we selected cemented carbide (WC-15% Co) on the DEFORM2D software mentioned earlier. The material constant of JIS SUS304, which is the work material, was determined from the flow stress–plastic strain curve obtained from the results of the performed tensile test shown in Figure 3. The damage value *C*, obtained from Cockcroft and Latham’s failure condition and expressed as Equation (1), was used to predict the fracture in shearing; in accordance with the previous report [26], this prediction was actually possible, where *C* is the damage value, *σ_max_* is the maximum principal stress, σ is the equivalent stress, and *ε* is the equivalent strain. In addition, according to the prediction, cracks and fractures that occurred during the blanking could be expressed using the element elimination method [23]; specifically, an element was eliminated when the damage value *C* of that element reached the fracture critical value *C_cr_* of 1.5 and an element with *C_cr_* of 1.5 was connected to four or more elements. The set critical value *C_cr_* of 1.5 is based on the fact that the fracture start was 78% of the plate thickness in the actual blanking experiment with a clearance *CL* = 2 μm The shear friction coefficient was assumed to be 0.08, referring to the value recommended by DEFORM2D for cemented carbide dies. Although there was concern that the friction may change significantly during the blanking process, as a result of examining them in the report of Sasada et al. [27], it was reported that the FEM analysis agrees well with the blanking result even if the friction coefficient was assumed to be constant.

(1) $$C=\int_{0}^{\bar{\varepsilon }}\frac{{\bar{\sigma }}_{max}}{\bar{\sigma }}d\bar{\varepsilon }$$

## 3. Results and Discussion

### 3.1. Results of Blanking Experiment

**Figure 4:**
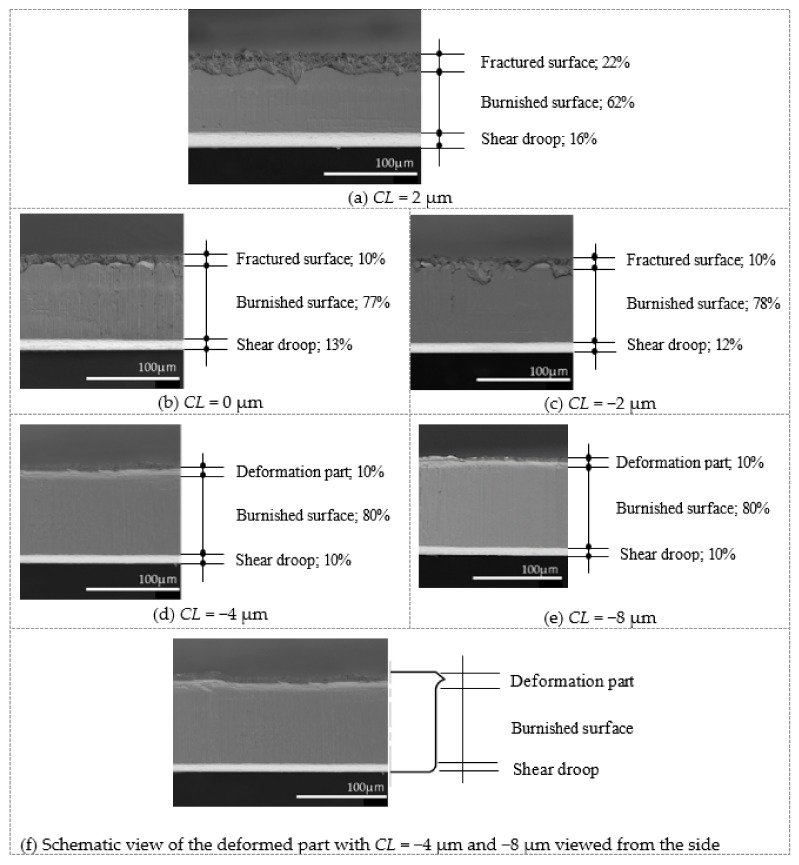
SEM image cut surface of various clearance blanking. (**a**) *CL* = 2 μm; 16% shear droop, 62% burnished surface, and 22% fractured surface, (**b**) *CL* = 0 μm; 13% shear droop, 77% burnished surface, and 10% fractured surface, (**c**) *CL* = −2 μm; 12% shear droop, 78% burnished surface, and 10% fractured surface, (**d**) *CL* = −4 μm; 10% shear droop, 80% burnished surface, and 10% deformation, (**e**) *CL* = −8 μm; 10% shear droop, 80% burnished surface, and 10% deformation, (**f**) Schematic view of the deformed part with *CL* = −4 μm and −8 μm viewed from the side.

First, Figure 4 shows SEM images of the cut surface of a blanked-out product at each clearance obtained in the blanking experiment. The percentage ratio of the cut surface at *CL* = 2 μm was about 16% shear droop, 62% burnished surface, and 22% fractured surface. At *CL* = 0 μm and −2 μm, the ratio of the shear droop and the fractured surface decreased, and the burnished surface ratio improved (*CL* = 0 μm: 13% shear droop, 77% burnished surface, and 10% fractured surface; *CL* = −2 μm: 12% shear droop, 78% burnished surface, and 10% fractured surface). At *CL* = −4 μm, the ratio of shear droop decreased further, and the fracture surface could not be confirmed, but, as shown in the schematic diagram of Figure 3f, shape deformation occurred along the die R (10% shear droop, 80% burnished surface, and 10% deformation). At *CL* = −8 μm and *CL* = −4 μm, the deformation values were the same (10% shear droop, 80% burnished surface, and 10% deformation). Incidentally, we assumed that these deformations in products and parts can be removed by the so-called barrel polishing, and products and parts can be used. Therefore, it was clarified that by adopting *CL* = −4 μm or −8 μm, it is possible to obtain parts with minimal shear droop and no fracture surface from the viewpoint of actual production.

**Figure 5:**
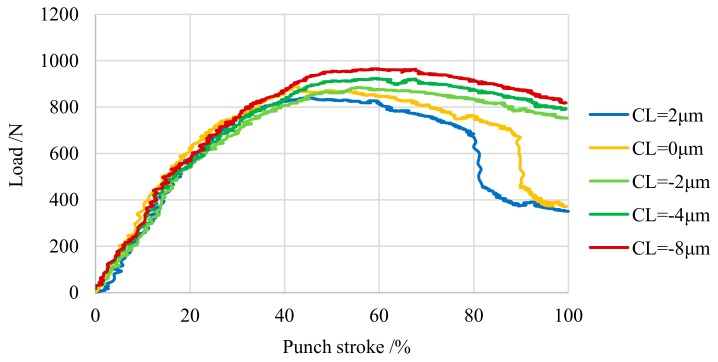
Load–stroke curves for various clearance.

Next, Figure 5 shows the blanking load stroke diagram obtained from the blanking experiment. The figure shows that the blanking load increases as clearance decreases. The shear energy obtained from the area of the load stroke diagram, which is a parameter representing the load applied to the punch, was increased by about 15% when *CL* = −8 μm compared to *CL* = 2 μm. A comparison of *CL* = −4 μm and −8 μm, at which the full-sheared product surface was obtained, showed that the blanking load and shear energy are lower at *CL* = −4 μm. Therefore, the optimum clearance obtained from the present blanking experiment is *CL* = −4 μm.

### 3.2. Consideration of Cut Surface Generation in Each Clearance by FEM Analysis

**Figure 6:**
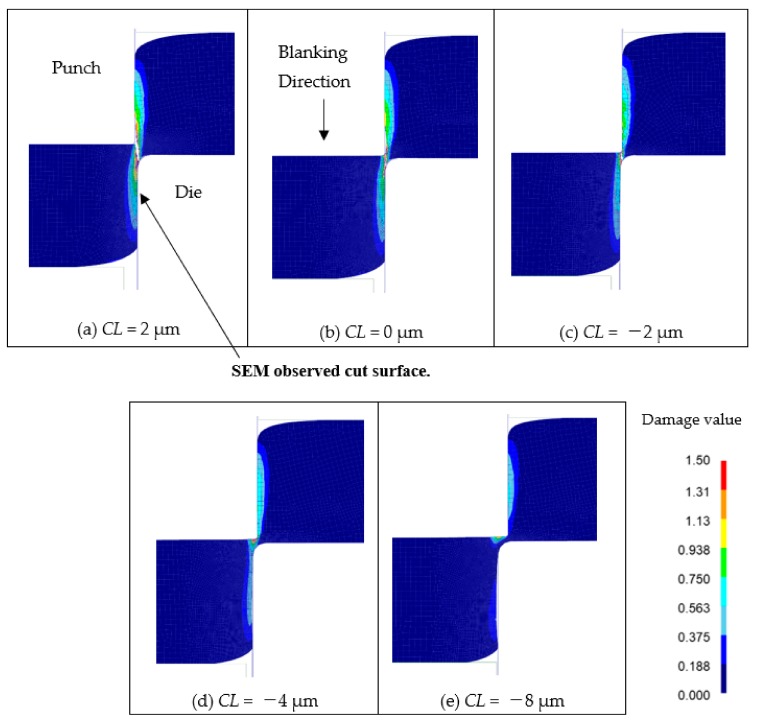
Comparison of damage value *C* of various clearance blanking. (**a**) *CL* = 2 μm, (**b**) *CL* = 0 μm, (**c**) *CL* = −2 μm, (**d**) *CL* = −4 μm, (**e**) *CL* = −8 μm.

Figure 6 shows the results of the cut surface at each clearance obtained by FEM analysis utilized damage value *C*. When the clearances were 2 μm, 0 μm, and −2 μm, the damage value *C* exceeded the critical fracture value *C_cr_* of 1.5, indicating that the elements were erased and cracks occurred, leading to fracture. It was simulated that no fracture at the product side occurred at *CL* = −4 μm and −8 μm. Furthermore, the results of FEM analysis for each clearance are very similar to those in the actual blanking experiment; here, the damage value *C* is obtained from the integral value along the strain history of the maximum principal stress, as shown in the previous Equation (1). That is, the damage value *C* increases as the tensile stress in shearing (the maximum principal stress is positive) increases. Therefore, to prevent the damage value *C* from becoming excessively large, it is necessary to prevent tensile stress from acting on the shearing region as much as possible. 

**Figure 7:**
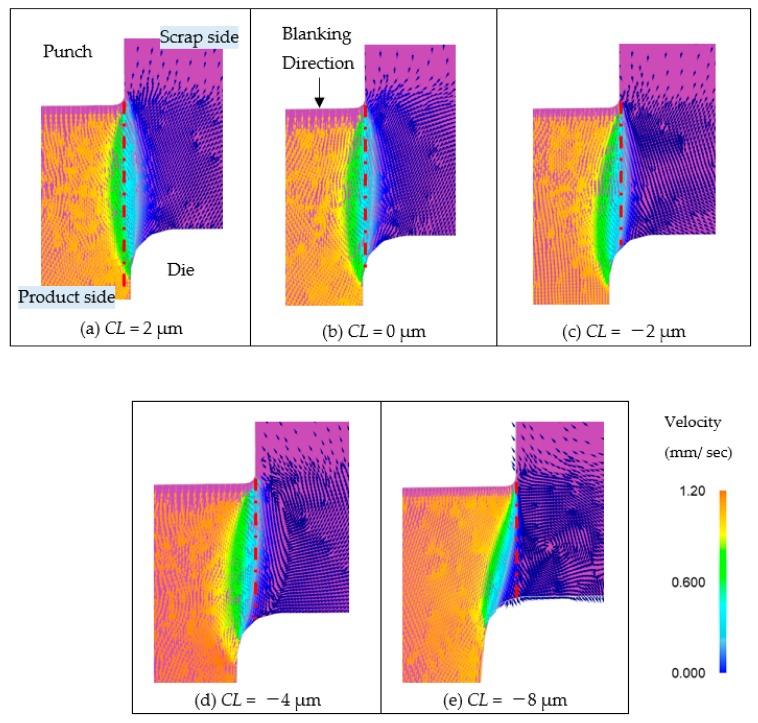
Comparison of material flow for 70%t punch penetration of various clearance blanking in enlarged view of shear deformation area. (**a**) *CL* = 2 μm, (**b**) *CL* = 0 μm, (**c**) *CL* = −2 μm, (**d**) *CL* = −4 μm, (**e**) *CL* = −8 μm.

Therefore, we evaluated and compared the flow states of materials that are considered to affect the stress state. Figure 7 is an enlarged view of the shear deformation area at the stage where the punch has penetrated 70% of the plate thickness, which is the step immediately before the fracture starts, and the difference in material flow at each clearance is shown by the flow velocity and flow direction. The single dot and dash line in a red color, shown in the figure, is the line drawn vertically from the punch tip, and becomes the path of the punch tip as the FEM analysis progresses. When *CL* = 2 μm, the velocity, along the single dot and dash line in a red color, is almost constant and the material flows smoothly. This means that the material of scrap is compressed by the blank holder and the material of product is pressed by the punch penetration. Then, the tensile stresses are generated in narrow shear deformation area. On the other hand, when the clearance goes to zero or negative, the velocity along the single dot and dash line in red color decreases as it approaches the die. Therefore, contrary to the case of *CL* = 2 μm, it is presumed that the tensile stress along the area is low.

**Figure 8:**
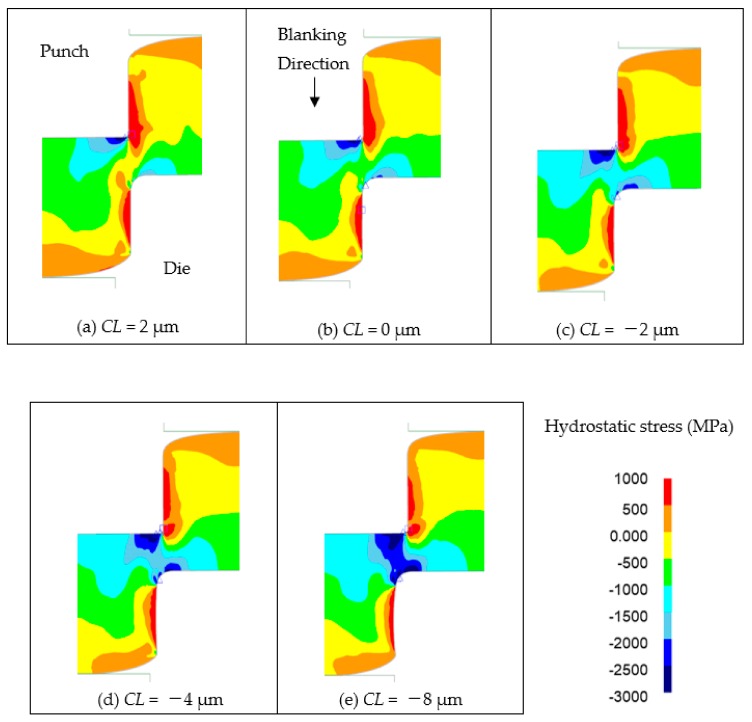
Comparison of hydrostatic stress for 70%t punch penetration of various clearance blanking. (**a**) *CL* = 2 μm, (**b**) *CL* = 0 μm, (**c**) *CL* = −2 μm, (**d**) *CL* = −4 μm, (**e**) *CL* = −8 μm.

Similar to the comparison of material flow, the hydrostatic stress should be also discussed. In general terms, the ductility of metals is said to improve under hydrostatic pressure [10]. However, the stress and strain results obtained by FEM analysis are calculated on the basis of the data obtained from the tensile test of the workpiece, so the metal ductility effect due to hydrostatic stress is not taken into account. However, as a guideline for die design in actual manufacturing, if the hydrostatic pressure state can be visualized, it may be useful to control the fracture surface ratio of the cut surface. Figure 8 shows the hydrostatic stress state of each clearance when the punch penetrated 70% of the plate thickness. As the clearance decreases, the hydrostatic stress decreases and the hydrostatic pressure increases. For example, hydrostatic stress value in the area connecting the punch corner and the die R corner were 0 MPa to −500 MPa at *CL* = 2 μm, −500 MPa to −1000 MPa at *CL* = 0 μm, −1000 MPa to −1500 MPa at *CL* = −2 μm, −1500 MPa to −2000 MPa at *CL* = −4 μm, and −2000 MPa to −2500 MPa at *CL* = −8 μm. It was shown that the clearance *CL*, at which the fracture does not occur in the above-mentioned blanking experiment, was −4 μm or less. The FEM analysis under the given conditions indicated that a hydrostatic stress of −1500 MPa or less is necessary. However, as mentioned above, the metal ductility effect of hydrostatic stress is not taken into account in the FEM analysis, so the relationship between the cut surface obtained in the punching experiment and the damage value in the FE analysis will be verified in the future. 

### 3.3. FEM Analysis of the Load on the Punch Tip

**Figure 9:**
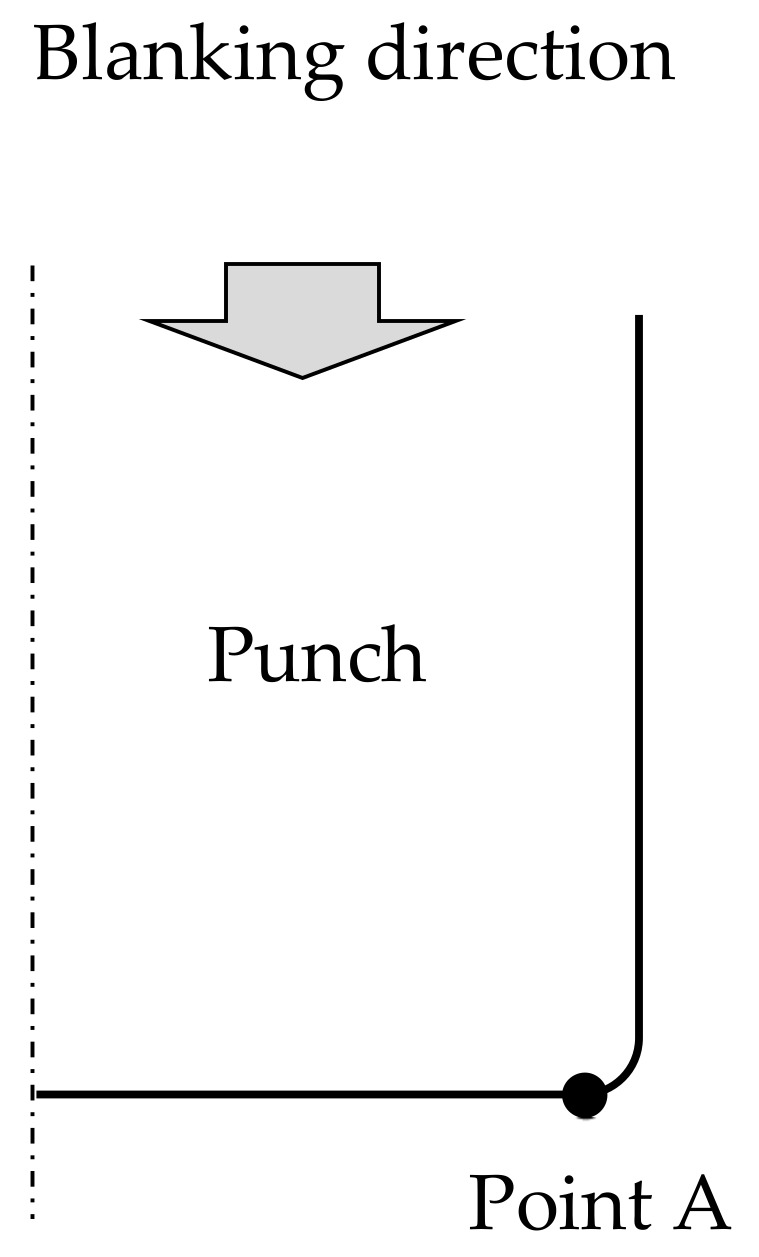
Stress estimation point in FEM analysis.

**Figure 10:**
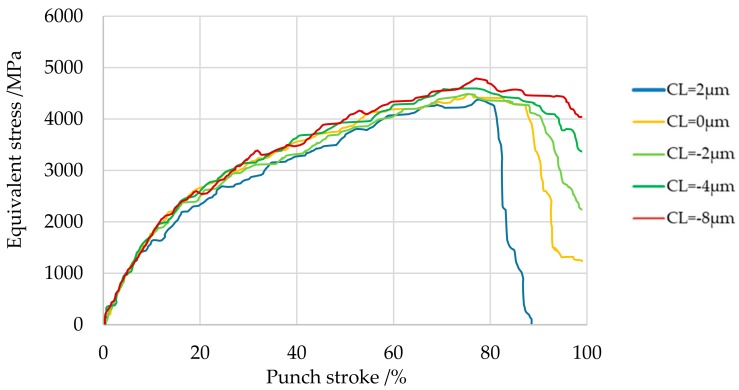
Equivalent stress at Point A at the tip of the punch for various clearances.

Generally, the punch breaks and wears at its tip during blanking [14]. Therefore, the relationship between clearance and the equivalent stress applied to the punch was evaluated at Point A at the start of the tip radius portion of the punch, as shown in Figure 9. Although not shown, Point A is the point with the highest equivalent stress in the equivalent stress distribution of the punch. Figure 10 shows the evaluation and comparison results of the aforementioned knowledge [14]. The equivalent stress increased as the clearance decreased, and the maximum equivalent stress was 4200 MPa at *CL* = 2 μm when punch stroke was about 80%. The maximum equivalent stress was highest at *CL* = −8 μm, reaching 4600 MPa when punch stroke was about 80%. Although not shown, the stress state was compression. In accordance with Table 2, which showed that fracture occurs when the compressive stress of cemented carbide reaches about 6880 MPa, in this FEM analysis, fracture compression loads of 61% and 67% were applied at *CL* = 2 μm and *CL* = −8 μm, respectively. To reduce the punch load, it is necessary to design dies and conditions such that the hydrostatic pressure stress is −1500 MPa even with a clearance *CL* = −4 μm or more. For example, the future challenge is to explore the possibility of reducing the punch load by examining the size and shape of the die R. The breakage and wear of the punch is most likely to occur when the punch returns from the workpiece [14]. Moreover, since the tensile strength is lower than the compressive strength of the cemented carbide punch [28], it is also extremely important to study the return process.

## 4. Conclusions

The effectiveness of negative clearance was verified from the viewpoint of the cut surface and punch load in micro component processing, where it is difficult to use so-called fine blanking. Specifically, a comprehensive blanking experiment and FEM analysis using a commercially available code were carried out, particularly from the viewpoint of the tool clearance conditions at narrow clearance, zero clearance, and negative clearance in the FB processing of the austenitic stainless steel JIS SUS304, and the following conclusions were obtained.

1. As the clearance decreases, the fractured surface of product side the cut surface decreases;
2. As the clearance decreases, the load on the punch tip increases;
3. *CL* = −4 μm is the optimum clearance to obtain a product side cut surface with no fracture and to reduce the punch load.

## Figures and Tables

**Table 1:** Mechanical properties of work material.

Tensile Strength (MPa)	896
0.2% Proof Stress (MPa)	583
Elongation (%)	47

**Table 2:** Material of the tools’ composition and mechanical properties.

Composition and Mechanical Properties	Punch	Die
Composition	WC-Co	WC-Co
Hardness (HRA)	95.0	91.5
Compressive Stress (MPa)	6880	5400

**Table 3:** Blanking tool specifications.

Items	Value
Clearance *CL* (μm)	2, 0, −2, −4, −8 (2%*t*, 0%*t*, −2%*t*, −4%*t*, −8%*t*)
Punch outer diameter *Dp_1_* (mm)	1.748
Die inner diameter *Dd_1_* (mm)	1.752, 1.748, 1.744, 1.740, 1.732
Punch outer diameter *Dp_2_* (mm)	1.740
Die inner diameter *Dd_2_* (mm)	1.750
Die radius *Rd*_1_ in 1st step (mm)	0.01
Die radius *Rd*_2_ in 2nd step (mm)	Nearly zero
Counterpunch outer diameter *Dc* (mm)	1.730
Blank holder force (*F_B_*)	500N (50% of blanking force)
Counterpunch force (*F_C_*)	200N (20% of blanking force)

**Table 4:** FEM simulation condition.

Simulation Model	Axisymmetric Model
Object type	Work material: elastic-plastic
	Punch/Die: elastic
	Blank holder/Stripper: rigid
	Counterpunch: rigid
Clearance *CL* (μm)	2, 0, −2, −4, −8, (2%*t*, 0%*t*, −2%*t*, −4%*t*, −8%*t*)
Punch outer diameter *Dp* (mm)	1.748
Die inner diameter *Dd* (mm)	1.752, 1.748, 1.744, 1.740, 1.732
Counterpunch outer diameter *Dc* (mm)	1.730
Work material outer diameter *Dw* (mm)	3.5
Tool cutting edges	*R_p_*= 0.002 mm, *R_d_*= 0.010 mm
Blank holder force (*F_B_*)	500 N (50% of blanking force)
Counterpunch force (*F_C_*)	200 N (20% of blanking force)
Blanked material	JIS SUS304 *t* = 0.1 mm Young’s modulus: 193 GPa Poisson’s ratio: 0.3
Ductile fracture criteria	Cockcroft–Latham
Fracture critical value *Ccr*	1.5
Shear friction coefficient (*μ*)	0.08
